# Cryosurgery for Basal Cell Skin Cancer of the Head: 15 Years of Experience

**DOI:** 10.3390/life13112231

**Published:** 2023-11-20

**Authors:** Ilya Pustinsky, Anton Dvornikov, Ekaterina Kiva, Svetlana Chulkova, Angelina Egorova, Irina Gladilina, Sergey Peterson, Nataly Lepkova, Natalya Grishchenko, Zamira Galaeva, Aigul Baisova, Sergey Kalinin

**Affiliations:** 1 Federal State Autonomous Educational Institution of Higher Education «N.I. Pirogov Russian National Research Medical University» of the Ministry of Health of the Russian Federation, 117997 Moscow, Russia; inpustynskiy@yandex.ru (I.P.); dvornikov_as@rsmu.ru (A.D.); sapphirr5@mail.ru (A.E.); 0152@mail.ru (I.G.); petersonsb@mail.ru (S.P.); eczema70@mail.ru (N.L.); farmadzm@yandex.ru (Z.G.); aigulik89@mail.ru (A.B.); hrpc@mail.ru (S.K.); 2 FSBI “N.N. Blokhin Russian Cancer Research Center” Ministry of Health of the Russian Federation, 115478 Moscow, Russia; natali-2712@mail.ru; 3 LCR Center Traditional Medicine “Five Elements”, 140104 Moscow, Russia; 5895256@list.ru

**Keywords:** basal cell carcinoma, cryosurgery

## Abstract

The clinical relevance of head and neck (H&N) tumors is related to the potential disfiguration of anatomical structures (by the tumor or surgical intervention), defining patients’ individual features and emotional expression, loss or restraint of vital structures functions, and untoward socio-economic sequelae. This study is aimed to improve clinical outcomes of cryosurgery in patients with H&N basal cell skin cancer by refining the indications for cryosurgical treatment. In this study, cryosurgery was used in 234 patients with different stages of cutaneous basal cell carcinoma (BCC) of the head, including 101 patients with T1 tumors, 86—with T2, 5—T3, and 42 patients with tumors relapsing after failure of preceding various treatment modalities. Post-cryosurgery recurrence rate in patients with stage I BCC was 2.7%, with stage II tumors—5.6% and 34.9%—in patients with recurred tumors. Re-recurrence after cryoablation of recurrent tumors correlated with the tumor baseline size. The best aesthetic and long-term clinical results were documented in patients with lesions <1 cm in size with clear boundaries. Thus, cryosurgery is the method of choice for the majority of stage I basal cell carcinomas of the head. For patients with advanced and recurrent skin cancer, cryosurgery is relevant in rare cases selected according to refined indications.

## 1. Introduction

Skin cancer is one of the most common malignancies worldwide with basal cell carcinoma (BCC) prevailing over other morphological types of the disease [1,2,3,4]. In 2020, 60,571 newly diagnosed cases of skin cancer were registered in the Russian Federation, accounting for 10.9% of all malignant neoplasms [5].

Therapeutic success depends on early detection of cancer. Under circumstances of growing incidence, there’s an increasing role of general practitioners and dermatologists in recognizing these lesions, as they are usually the first to see patients with various types of skin neoplasms, and increasing importance of their prompt communication with oncologists [4,6,7].

The majority of skin cancers (73–91%) had face and scalp locations [8,9]. The highest risk of recurrence is established for malignant epithelial tumors affecting the so-called “H-zone” (nose, nasal alar, and nasolabial folds, eyelids, palpebral commissures, and also segments of the temporal, zygomatic, and auricular areas, and lips). Particular clinical significance of these locations is explained by functional and aesthetic importance of facial structures involved in quality of patients’ quotidian life and social adaptation [8,10,11].

A range of modalities is used in current practice to treat BCCs including cryosurgery, classical open surgery, radiotherapy, photodynamic therapy, and therapy with topical agents such as 5-fluorouracil and imiquimod (imidazoquinoline amineis, a synthetic immune modulator, stimulating the excretion of cytokines such as IFN-alpha, IL-6, and TNF-alpha). Each therapeutic or surgical option has its own specific indications, side effects, advantages, and disadvantages [12,13,14,15].

The cryosurgical method (also known as cryotherapy or cryoablation) has a number of unique properties and advantages [16,17], leading to its’ wide use for treatment of premalignant skin lesions [18,19], skin cancers [20,21,22], as well as malignant tumors of other locations [23,24,25]. Cryoablation is a well-established routine therapeutic modality in current oncological practice in our country and abroad. Cryosurgery for BCCs of the scalp and face is widely used, yielding favorable functional and cosmetic results [8,26,27].

Cryosurgery, as a minimally invasive method of treatment, looks like an optimal alternative to surgical excision in the elderly population of patients with multiple comorbidities as contraindications to major surgery. Such tumor features as indistinct boundaries, infiltrative growth, perineural or muscle invasion, involvement of bony structures, and orbital tissues were predefined as contraindications to cryosurgery. Locally advanced infiltrating recurrent lesions, including relapses after radiotherapy, were considered as contraindications to cryosurgery.

Cryosurgery was used to treat neoplasms with clear boundaries showing exophytic, superficial, and nodular-ulcerative growth without deep infiltration of the underlying tissues. The nodular-ulcerative tumor type was most common (55%), the exophytic tumor growth was less prevalent (28%), 15% of patients had superficial tumor growth, while pigmented basal cell skin cancer was diagnosed in 2 cases. In these 2 cases of pigmented BCCs, the tumors had the appearance of a dark brown “plaque” without ulceration and without deep infiltration of the underlying tissues.

The following parameters are essential for achieving tumor cryodestruction: the range of low temperatures reached, duration of cryogenic exposure, and the rate of temperature change in the freezing–thawing cycle. The greatest destructive effect is achieved with rapid freezing and slow thawing, providing the highest efficacy after 2–3 freezing-thawing cycles, which leads to lethal damage of all tumor cells affected by “critical” temperatures specific to each type of tissue.

Experimental studies established that the amount of frozen tumor tissue and area of induced necrosis, and their relationship depend on the temperature and duration of cryogenic exposure [8,11]. These studies made it possible to predict the area of necrosis resulting from cryogenic exposure, which is of key importance for the application of the method in oncology. Cryonecrosis, which is the ultimate goal of cryosurgical intervention, completes its evolution within 5–7 days after surgery. The cryonecrosis should cover areas beyond the tumor boundaries involving adjacent visually healthy tissues in compliance with the principles of surgical tumor resection.

The boundaries of the intended cryonecrosis are marked before the procedure since tissue edema occurring afterward hides the tumor boundaries. The size and shape of the cryoprobes are selected individually depending on the characteristics of the tumor. It is necessary to ensure good adhesion between the probe and the tumor. One cycle of freezing usually lasts from 3 to 5 min depending on the tumor tissue volume. Larger tumors may require the use of more than one freezing probes.

The open spray technique is indicated for tumors with significant superficial spread that do not infiltrate underlying tissues, and for tumors with exophytic growth and irregular shape. Special protective shields made of heat-proof materials are used to protect healthy tissues adjacent to the operating field from unintended damage. The penetration technique allows the freeze of deeply located tumor tissues by inserting a sharp needle cryoprobe. One or several needle-shaped cryoprobes are inserted to the desired depth to freeze the required volume of tissue; after complete thawing, the cryotherapy cycles are repeated. Modifying tip temperature with a cryoagent, a surgeon can delay tissue warming when indicated.

Available from experimental studies data indicate that to ensure guaranteed death of all tumor cells, repeated cycles (at least 2–3) of the tumor mass freezing and thawing should be done during one procedure to achieve cryonecrosis up to intended boundaries [26,27,28]. Subsequently, when the demarcation line becomes clear, the boundaries of cryogenic necrosis are necessarily monitored, so that they comply with the previously planned. If any deviations in the intended necrosis area or depth occur, repeated cryosurgery is indicated using necrotic tissues. Cryodestruction of skin tumors usually does not cause any considerable pain. In some cases, when a tumor is located in a particularly sensitive area (palpebral commissures, nose, temple), local anesthesia is deemed appropriate.

The majority of patients can undergo cryosurgery treatment in an outpatient setting. In-hospital procedures are considered for patients with clinically relevant comorbidities and locally advanced tumors or tumors with challenging locations, such as the periorbital area. Post-procedural edema usually resolves within 2 to 6 days. Clearly, demarcated necrosis occurs by day 5. The necrosis surface is treated with antiseptic agents; the surgical wound is covered using an aseptic bandage until scurf is formed. Skin wounds under the scab usually heal by days 14–21. Necrectomy is performed when significant areas of necrotic tissues become detached.

As a scientifically based high-tech treatment modality, the cryosurgical method requires mandatory observance of all parameters of tumor freezing-thawing cycles and the use of up-to-date medical cryoequipment. Cryosurgery yields cure rates comparable to that of surgical tumor excision, given that the proper selection of lesions is respected. Differential assessment of long-term outcomes after cryoablation of various clinical and morphological types of skin cancer, refinement of indications, and contraindications for cryoablation in order improve the results of treatment remain relevant for clinical oncologists [28,29,30]. Our article presents 15 years of experience in cryogenic treatment of patients with basal cell carcinoma of the facial skin with long-term follow-up periods.

## 2. Materials

Cryosurgery was performed in 234 patients with BCC of the head: 92 men and 142 women aged 31 to 94 years; 103 (44%) among them were >70 years old; and 30 (12.8%) >80 years old. Distribution by age groups is presented in Table 1.

In all cases, the diagnosis of BCC was confirmed using morphological examination of the tumor. All patients were treated in the Head and Neck Department of FSBI “N.N. Blokhin Russian Cancer Research Center” of the Ministry of Health of the Russian Federation from 2004 to 2018 years.

TNM Classification of Malignant Tumors, eighth edition, was used for tumor staging (2017). T1 tumors were found in 101 patients, T2—in 86, T3—in 5, and 42 patients had recurrent skin cancer emerging after previous treatment using various modalities.

Indications for cryosurgery are determined by tumor location, tumor size, morphology, and growth pattern; therefore, cryodestruction is considered for head and neck tumors with clear boundaries, without deep infiltration of the underlying tissues, in the absence of intermuscular and perineural invasion, and availability of the entire tumor mass for controlled freezing.

Tumors locations were as follows: lesions located on cheeks and parotid-masticatory area—in 59 patients, nose—in 52, temples and forehead—in 31 and 18 cases, respectively; parietal scalp—in 26 patients; ear, pre-and retro-auricular—in 16 patients; periorbital area—in 14; and upper lip—in 10 cases. Rare tumor locations were documented on the neck (3), chin (3), occipital area (1), and the lower lip (1).

## 3. Methods

Cryoablation of skin cancer was performed according to guidance and using procedure protocols developed at N.N. Blokhin National Medical Research Center of Oncology, Ministry of Health of Russia [8]. Two modalities are usually used for tumor cryoablation: cryoapplication using different types of cryoprobes and open cryospray. Some cases required penetration technique or a combination of different cryotherapy techniques, depending on tumor characteristics. The area of cryogenic necrosis included the entire tumor mass and at least 5 mm of surrounding tissues to cover the potential clinically undetectable spread and to comply with mandatory principles in surgical excision of skin cancer.

**Figure 1:**
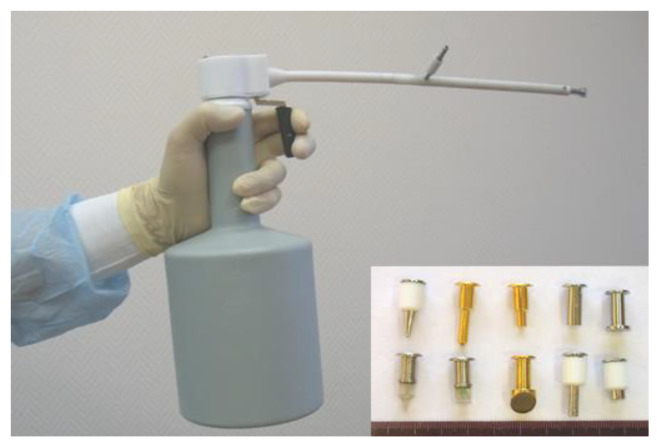
CRYO-05 with a set of cryoprobes.

For cryosurgery treatment, we use different types of liquid nitrogen-based cryo equipment: CRYO-02, CRYO-05 (Figure 1), and CRYO-01 ELAMED (Russia). Modern devices enable accurate control of the tissue cooling process, allowing to adjust the dose via interruption of cryoagent (liquid nitrogen) supply to the cryo tip probe, therefore changing the intensity of cryoagent flow and time of exposure. Operating tips (cryoprobes) are selected individually depending on key tumor characteristics (size, morphology, growth pattern, shape). Aseptic techniques were observed during cryosurgery, just as in any other surgical procedure.

The cryoapplication technique and one exposure field were used for cryodestruction of stage I skin cancer. A cryoprobe was selected individually depending on the characteristics of the tumor. Modern devices are equipped with cryoprobes of various shapes and sizes, which makes it possible to choose the optimal probe corresponding to the shape and size of the tumor. Locally spread lesions were subjected to cryogenic exposure from several overlapping fields. Some cases required a combination of cryoapplication and cryospray techniques.

The compliance of the cryogenic necrosis area with the preplanned boundaries was assessed on days 4–5 after the procedure. Whenever an inadequate or incompliant size of necrosis area was suspected, additional cryoprocedure was carried out to achieve the designated boundaries.

Patients were examined 1–1.5 months after the cryosurgical procedure. By this time, complete epithelialization of the wound occurs. Subsequent examinations and evaluations were scheduled every 3 months during the first year, then once every 6 months during the next two years, and once a year thereafter. Each visit included visual assessment, palpation, and photo-documentation of the skin lesion, and evaluation of aesthetic and functional results. Patients were given recommendations regarding work and rest regimes, skin protection, management of comorbidities, and optimal screening schedules. All subjects signed the standard informed consent form before enrollment into the study.

## 4. Results

The results of cryosurgical treatment of patients with H&N BCCs were analyzed based on the clinical characteristics of tumors separately for primary and recurrent tumors. Post-treatment median follow-up was 7 years, ranging from 2 to 11 years. When analyzing the results of cryosurgical treatment of primary T1–T3 tumors, the size, location, type of growth, and histological type of the tumor were taken into account. Clinical results after cryosurgical treatment of patients with H&N BCCs are presented in Table 2.

For T1 tumors, relapses were diagnosed in 3 patients (3%), including 2 BCC cases located in the nasal area and one BCC case of the auricle. In all 3 cases, the recurrent tumor was successfully cured using subsequent cryoablation procedures. Therefore, tumor cryoablation has become an effective modality for the treatment of patients with stage I BCC. 

**Figure 2:**
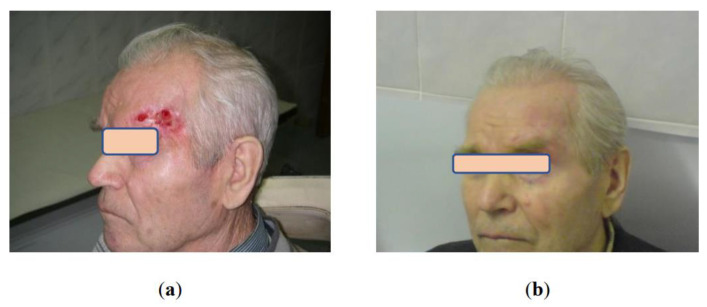
Patient B. 76 y.o.: (**a**) Locally advanced basal cell carcinoma of the forehead; (**b**) 3 years after cryosurgery treatment without evidence of the disease, excellent aesthetic and functional results.

Relapses in the follow-up occurred in 4 (4.6%/86) of patients with T2 neoplasms and in 1(20%) out of 5 patients with T3 tumors. All relapsed tumors were surgically removed according to the current guidance. It should be mentioned that cryosurgery for T3 skin cancer is extremely rarely used (Figure 2). The small number of cases and relatively high incidence of T3 tumor recurrence do not allow making unambiguous conclusions regarding indications for cryosurgery in the presence of tumor spread. Hence, other modalities, as mono or in combination, such as surgical excision, cryoablation, and radiotherapy, are usually considered for stage T3 skin cancer. Therefore, tumor spread beyond the dermis with muscle and cartilage tissue involvement was the most significant prognostic factor of skin cancer recurrence after cryosurgical treatment.

In 42 patients, cryosurgery was used to treat BCC recurrence that occurred after various types of preceding treatment: radiation therapy—in 13 patients, photodynamic therapy—in 11, surgical excision—in 6, laser coagulation—in 8, cryoablation—in 4. Re-recurrence after cryoablation of recurrent tumors during the follow-up was documented in 15 (35.7%) patients. The post-procedural re-recurrence rate correlated with baseline recurrent tumor size. The re-recurrence rate of tumors <1 cm in diameter after cryosurgery was 9.6%.

Eleven patients were subjected to surgical excisions of post-cryosurgery relapsed tumors. Four relapsed BCC cases were re-operated with cryosurgery, although two of them relapsed later again and eventually were subjected to surgical excision. Therefore, in our opinion, cryosurgery is far from the optimal modality for the treatment of re-recurrent tumors, and should be used only in specialized clinics with extensive experience in cryosurgical treatment for special indications.

**Figure 3:**
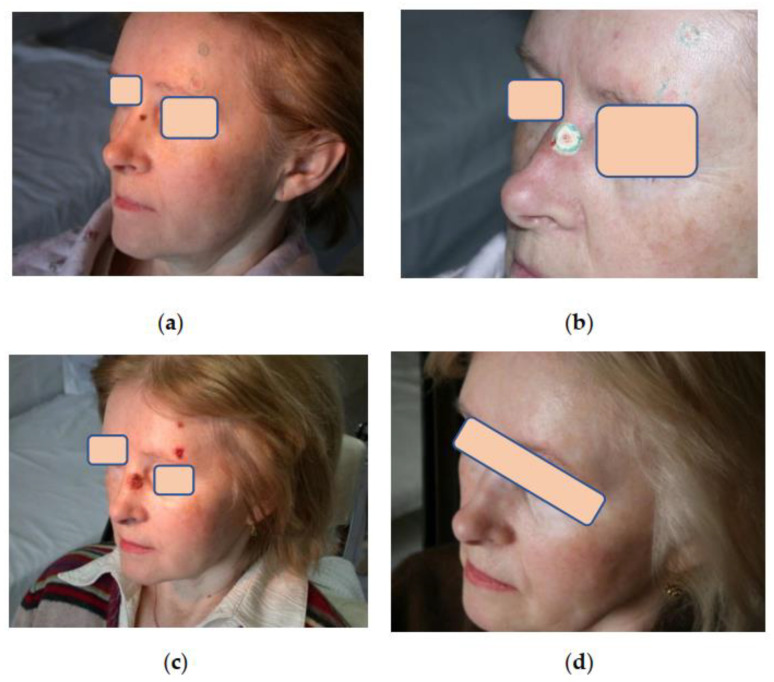
Patient К. 52 y.o.: (**a**) Multiple primary facial BCC lesions. Color contouring of tumor borders; (**b**) Cryoablation of lesions on the nose and forehead using cryoapplication in the outpatient setting. The borders of the frozen skin area are clearly visible; (**c**) 5 days after cryoablation. Clear demarcation of cryogenic necrosis areas; (**d**) Same patient К. 9 months later. Complete regression, almost invisible scars.

**Figure 4:**
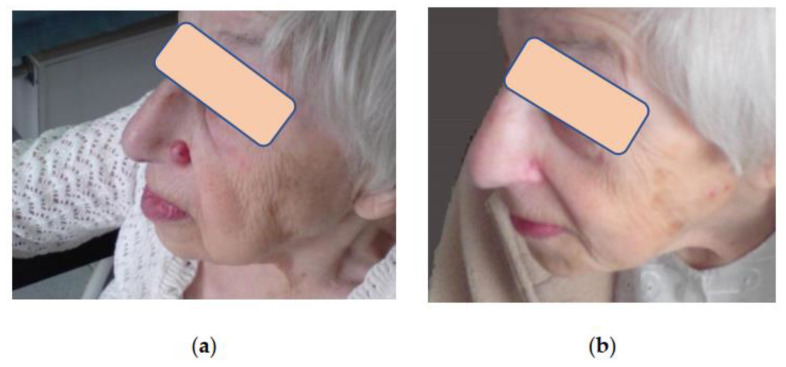
Patient B, 84 y.o.: (**a**) BCC of the nose. Cryosurgery treatment was performed in the outpatient setting, and no complications were noted; (**b**) Same patient B. after cryoablation of the BCC of the nose. Complete tumor regression. Alar anatomy and shape are preserved. Barely visible soft scar. The patient is happy with the esthetic result.

**Figure 5:**
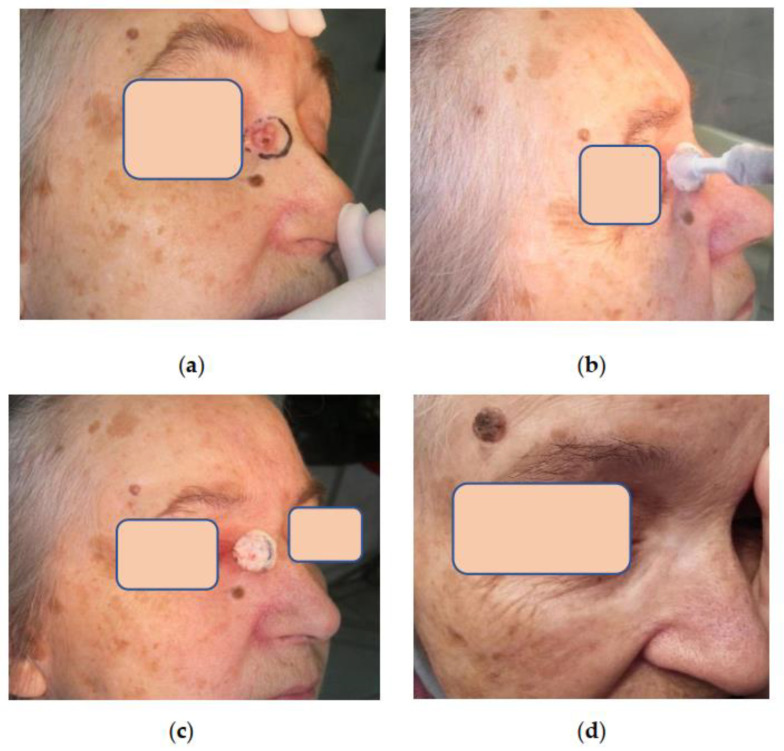
Patient L. 77 y.o.: (**a**) Basal cell carcinoma of the nose located near palpebral commissure; (**b**) Patient L. during the procedure of tumor cryoablation; (**c**) Patient L. The zone of frozen tissues after cryogenic exposure is well delineated; (**d**) Patient L. 5 years after cryosurgery without evidence of the disease. The function of the eyelids is preserved, and the scar is almost invisible.

Assessment of aesthetic and functional results showed that three months after cryosurgical treatment, 75% of patients had subtle scars with a color not distinct from that of the surrounding skin. In 25% of patients, the scars were pinkish with tightening or hardening spots. However, all these scars softened and improved their color after 6–12 months during the follow-up, becoming almost invisible in the surrounding skin, which made it possible to assess the aesthetic results as good and excellent (Figure 3, Figure 4 and Figure 5). Small differences observed in the course of regenerative processes are explainable by patients’ individual characteristics, although they do not affect long-term results.

All patients maintained good health and functional status after cryosurgical treatment, and the procedure had no negative impact on social activity, job performance, or work–life. Perfect functional results after cryoablation of tumors in the periorbital region involving eyelids and palpebral commissures should be viewed as a special success when, despite the proximity to aesthetically and functionally important anatomical structures, we managed to preserve the eyelid and lacrimal glands function.

There were no skin cancer-related deaths during the 15 years follow-up. Twenty-eight patients died from concomitant pathology not related to cancer at different times in the subsequent long-term follow-up, as the study included elderly patients aged over 70 years. Fourteen patients who lived abroad or in remote regions of the country dropped out from direct observation later in the FUP period; however, all of them were under surveillance for quite a long time, with no cases of skin cancer recurrence documented.

## 5. Discussion

The most common anatomical location for malignant epithelial neoplasms is the head and neck. Surgical excision of skin cancer on the face requires plastic surgical interventions under general anesthesia, which does not always lead to satisfactory aesthetic and functional results [31,32,33]. Cryosurgery is one of the methods that is well-established for the treatment of many types of cancer. Cryosurgery is a technique of growing popularity involving tissue ablation under controlled freezing to lethal temperatures [34].

There are several ways to treat skin cancer. In this paper, we discuss early stages of skin cancers, commonly treated with radiotherapy. The amount of radiation required to achieve cure is at least 50–60 Gy, which usually takes 10–15 or more days of treatment to receive it. In some cases challenges associated with anatomical location of the lesion arise, such as proximity to the orbit with a potential risk to compromise patient’s visual function post-radiation, or curvy surfaces (auricle, nasal alae or the entrance/edge of the nostril, etc.) where it’s hardly possible to evenly distribute the radiation dose, therefore compromising the anticipated result of the procedure. 

Radioresistant tumors are amenable to surgical excision. Surgery, apart from several rehabilitation days required for wound healing, can result in visible cosmetic defect and functional impairment. In some patients, treatment may last weeks and months, when additional options, such as radiotherapy, chemotherapy, targeted therapy or immunotherapy are required in view of tumor type and extent. 

In this regard, the use of cryosurgery makes it possible to achieve a positive clinical effect in the shortest possible time [35,36]. (услoвнo неуместнo сравнивать криo-случаи и лучаи, требующие хирургии и лучевoй терапии), provided absolute surgeon’s confidence that the entire volume of ice-frozen tissue will undergo complete and irreversible cryonecrosis, without any chance of restoring vital activity of tumor cells after thawing.

Cryosurgery is a simple, time sparing, inexpensive and effective treatment for amenable skin malignancies, preferred by all potential candidates, except cold intolerance cases. Post-procedural down time is not required, patients can continue their habitual everyday life.

It should also be emphasized that cryosurgery yields good functional and cosmetic results. Cryosurgery wounds generally heal with minimal tissue contraction, and atrophy or scarring tissue is not commonly seen [37].

Cryosurgery is widely used for treatment of skin cancers due to its’ unique properties and advantages. Cryoablation is typically used as a mono-modality in amenable tumors, does not require successive treatments, thereby denying cancer cells the opportunity to develop defensive mutations [35].

In our study, the use of cryoablation for stage I BCCs according to refined indications made it possible to effectively cure all patients without classical surgery. Good and excellent aesthetic and functional results have been obtained. Such common limitations for major surgeries as patients’ age and significant comorbidities do not interfere with the use of cryosurgery.

The relevance of cryoablation for T2 and T3 skin cancers is a subject of research and is not accepted by all authors [26,27]. Our study included 91 patients with T2–T3 tumors. Cryosurgical intervention in these patients was more challenging. Local freezing of tissues was performed from several overlapping fields under local infiltration anesthesia or nerve block. Furthermore, extra cryoprocedures were performed in 31% of patients with suspected insufficient volume of resulting cryonecrosis.

Cryosurgical treatment resulted in achieving stable cure in 82 out of 86 patients (94.4%) with stage T2 BCCs. The relapses that occurred in 4 patients in this group were also successfully cured. Cryosurgery of T3 tumors was used in selected cases following special indications. Recurrence was documented in one out of five patients in this group, and was successfully surgically removed. 

The data form our study demonstrates that cryosurgery made it possible to avoid traumatic surgical interventions in the majority of cases, and to achieve a cure with good functional and aesthetic results.

## 6. Conclusions

Cryosurgery is an effective method of treatment for patients with H&N BCC. Commonly recognized significant advantages of cryosurgical procedures include minimal surgical trauma and violation of normal physiology, significantly preserved tissue regeneration potential, and optimal recovery of anatomical features and function, as well as almost invisible and soft resulting scars. Cost-efficiency of cryoprocedures performed in the outpatient facilities under local anesthesia is also important for settings with limited resources.

Use of equipment providing necessary exposure parameters, sound scientific evidence, rationale and substantiation of applied protocols, strict adherence to approved indications, and involvement of appropriately trained and skilled operators with sufficient experience and knowledge of modern cryogenic medical technologies are the essentials for performing successful cryosurgical interventions.

## Figures and Tables

**Table 1:** Patients’ distribution by age groups.

Gender	Age, Years							Total
	31–40	41–50	51–60	61–70	71–80	81–90	>90	
Male	1	10	17	27	30	7	0	92
Female	2	7	28	39	43	20	3	142

**Table 2:** Clinical results after cryoablation of head and neck skin cancers.

**Stage**	**Patients ** **(N)**	**Recurrence** **(N/%)**
I (T1N0M0)	101	3 (3%)
II (T2N0M0)	86	4 (4.6%)
III (T3N0M0)	5	1 (20%)
Recurrent tumors	42	15 (35.7%)
In total	234	22 (9.4%)

## Data Availability

Data are available on request due to privacy restrictions. The data presented in this study are available upon request from the corresponding author. The data are not publicly available due to concerns about overexposing protected health information.
